# Supplementary material for: Causal interplay between lactose intolerance and gut microbiota: a combined bidirectional Mendelian randomization and in vivo validation study
Source: Front Nutr. 2026 Jun 1;13:1803337. doi: 10.3389/fnut.2026.1803337 (PMC13265576; doi:10.3389/fnut.2026.1803337)
Supplement: Supplementary file 1 [file Data_Sheet_1.zip › supplementary materials/Forward/leave one out cross validation/ebi-a-GCST90027560.finngen_R12_E4_LACTONAS.pdf]

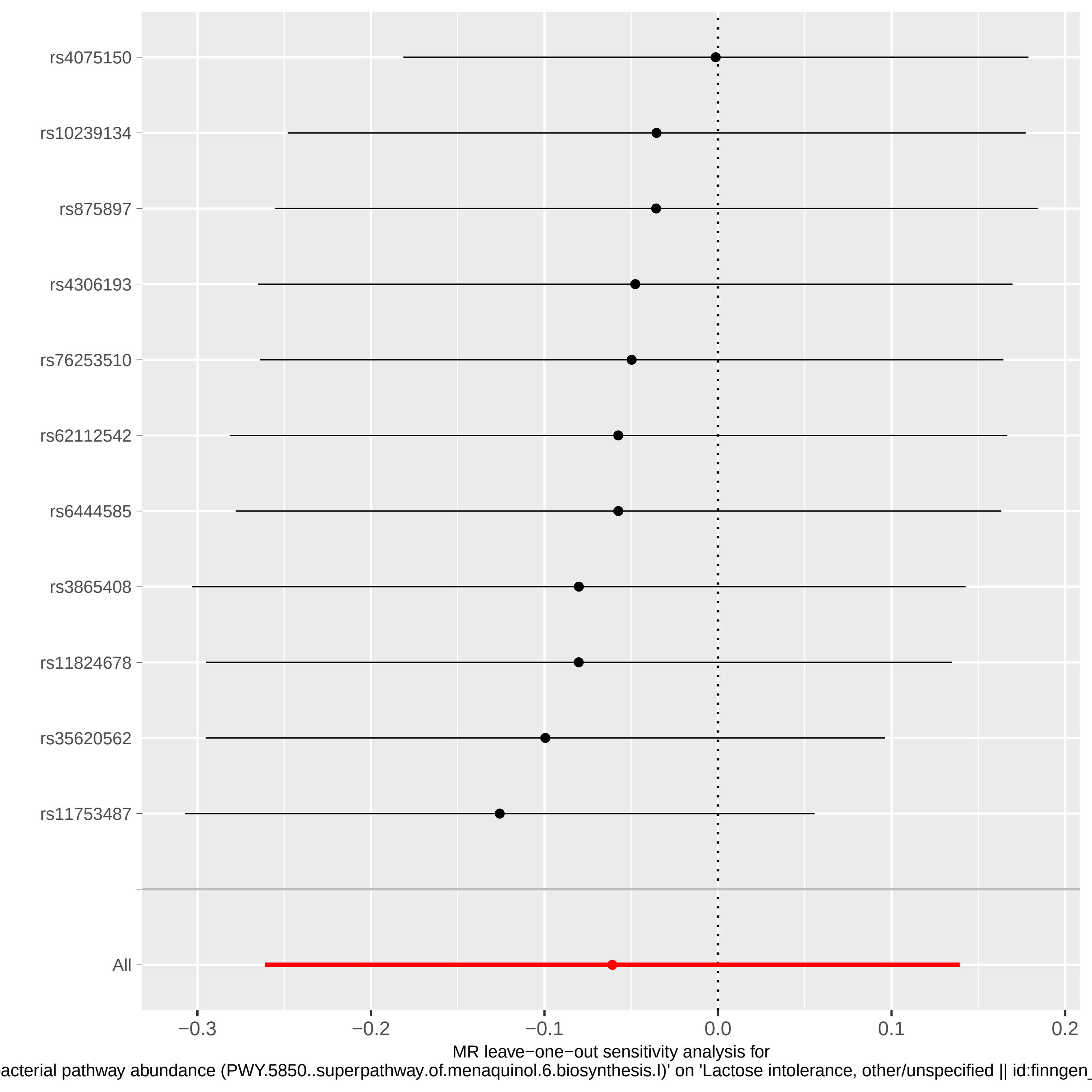

rs4075150
rs10239134
rs875897
rs4306193
rs76253510
rs62112542
rs6444585
rs3865408
rs11824678
rs35620562
rs11753487
All
−0.3
−0.2
−0.1
0.0
0.1
0.2
MR leave−one−out sensitivity analysis for
acterial pathway abundance (PWY.5850..superpathway.of.menaquinol.6.biosynthesis.I)' on 'Lactose intolerance, other/unspecified || id:finngen_
